# Supplementary material for: Structure-Function Studies of Polymyxin B Lipononapeptides
Source: Molecules. 2019 Feb 2;24(3):553. doi: 10.3390/molecules24030553 (PMC6384738; doi:10.3390/molecules24030553)
Supplement: Supplementary file 1 [file molecules-24-00553-s001.pdf]

## Supporting Information

# Structure-Function Studies of Polymyxin B Lipononapeptides

**Alejandra Gallardo-Godoy, Karl A. Hansford *, Craig Muldoon, Bernd Becker, Alysha G. Elliott, Johnny X. Huang, Ruby Pelingon, Mark S. Butler, Mark A. T. Blaskovich * and Matthew A. Cooper ***

Institute for Molecular Bioscience, The University of Queensland, Brisbane, Queensland 4072, Australia; a.gallardogodoy@uq.edu.au (A.G.-G.); cmouldy@yahoo.com.au (C.M.); bbeckerr@gmail.com (B.B.); a.elliott@imb.uq.edu.au (A.G.E.); johnny.xiao.huang@gmail.com (J.X.H.); r.pelingon@imb.uq.edu.au (R.P.); m.butler5@uq.edu.au (M.S.B.)

* Correspondence: k.hansford@uq.edu.au (K.A.H.); m.blaskovich@uq.edu.au (M.A.T.B.); m.cooper@uq.edu.au (M.A.C.); Tel.: +61-7-3346-2107 (K.A.H).

**Table S2.** Bacterial strains used for Minimum Inhibitory Concentration (MIC) determinations.

| Organism | Strain | Strain description | Strain Source |
|---|---|---|---|
| ***Escherichia coli*** | ATCC 25922 | FDA strain Seattle 1946 | ATCC |
| ***Klebsiella pneumoniae*** | ATCC 13883 | Control strain | ATCC |
| ***Klebsiella pneumoniae**** | ATCC 700603 | K6; ESBL (Extended spectrum ß-lactamase) producer SHV-18 | ATCC |
| ***Klebsiella pneumoniae*** | BAA-2146 | NDM-1 (New Delhi Metallo-beta-lactamase-1) positive | ATCC |
| ***Acinetobacter baumannii*** | ATCC 19606 | Type strain | ATCC |
| ***Pseudomonas aeruginosa*** | ATCC 27853 | Quality control strain | ATCC |
| ***Pseudomonas aeruginosa*** | FADDI-PA070 | Clinical isolate, polymyxin resistant | Nation and Li labs, Monash University |
| ***Staphylococcus aureus*** | ATCC 25923 | MSSA (methicillin Susceptible *S. aureus*) | ATCC |

**Recently suggested for reclassification as K. quasipneumoniae subsp. similipneumoniae[1, 2]*

**Scheme S1.** General on-resin cyclisation synthetic route to polymyxin nonapeptides, exemplified by synthesis of PMBN, compound **8** and compound **20**[a]

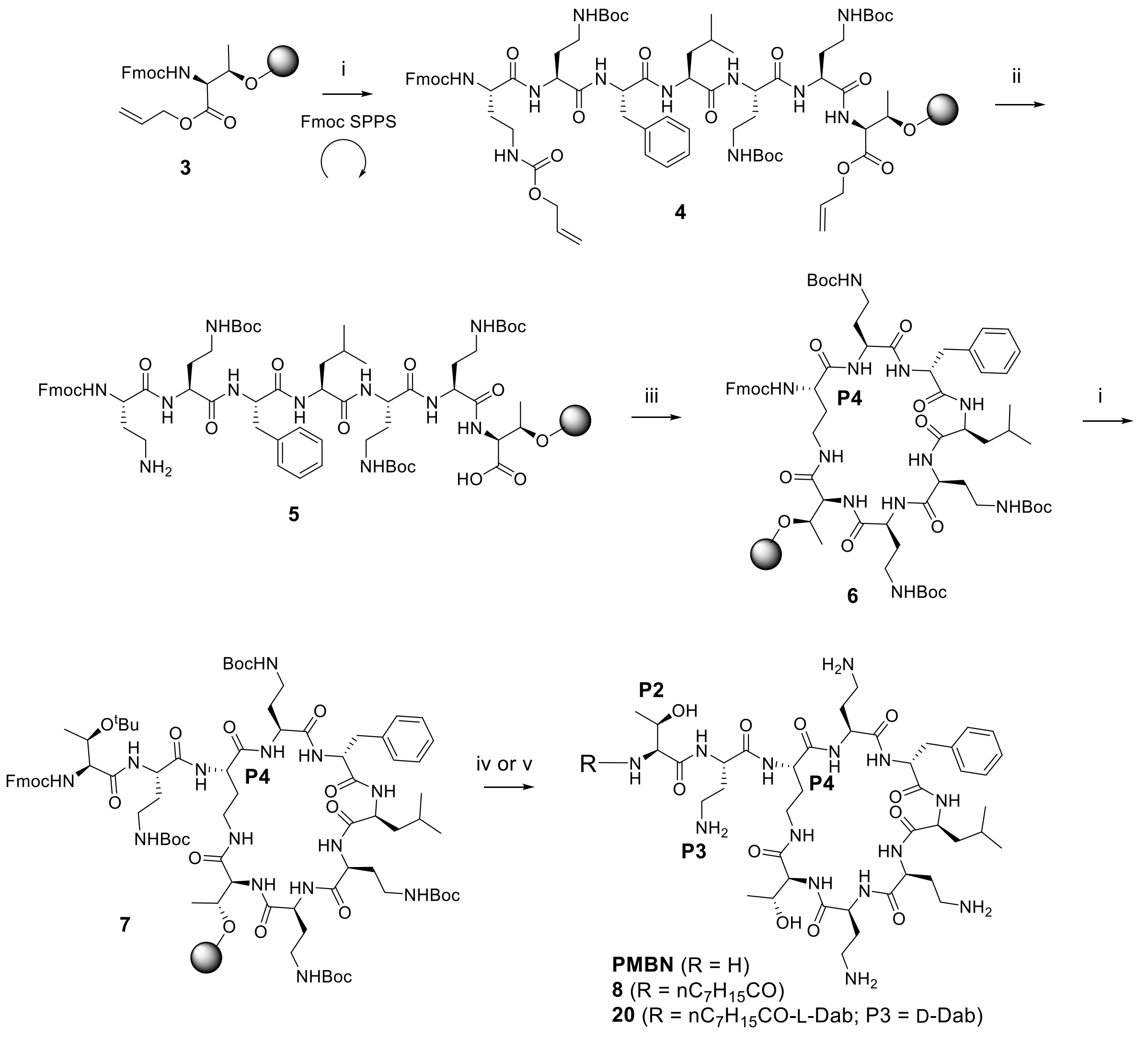

[a]Reagent and conditions: (i) (a) 30% piperidine, DMF; (b) Fmoc-amino acid, HCTU, DIPEA, DMF; (c) repeat; (ii) $Pd(PPh_3)_4$, $PhSiH_3$; (iii) DPPA, DIPEA, DMF; (iv) (a) 30% piperidine, DMF; (b) $TFA/Et_3SiH/H_2O$ (95:1:4); (v) (a) 30% piperidine, DMF; (b) octanoic acid, HCTU, DIPEA, DMF; (c) $TFA/Et_3SiH/H_2O$ (95:1:4).

**Materials and Methods: Synthesis**

All chemicals were obtained from commercial suppliers and used without further purification. All Fmoc α-amino acids and *O*-(1H-6-chlorobenzotriazole-1-yl)-1,1,3,3-tetramethyluronium hexafluorophosphate (HCTU) were purchased from Chem-Impex International Inc. (Wood Dale, IL, USA). 3,4-Dihydro-2H-pyran-2-yl-methoxymethyl polystyrene resin (DHP HM resin 100 – 200 mesh) was purchased from Novabiochem (Merck). Peptide grade trifluoroacetic acid (TFA), piperidine, methanol and *N,N*-dimethylformamide (DMF) were purchased from AusPep (Melbourne, Australia). All other reagents, including gentamicin sulfate (G1914), polymyxin B sulfate (P0972) and vancomycin hydrochloride hydrate (861987) were purchased from Sigma-Aldrich (Sydney, Australia). LC-MS analyses were conducted using Agilent Technologies 1200 Series Instrument with a G1316A variable wavelength detector set at λ= 210 nm, 1200 Series ELSD, 6110 quadrupole ESI-MS, using an Agilent Eclipse XDB-Phenyl column (3 × 100mm, 3.5 µm particle size, flow rate 1 mL/min, the mobile phases 0.05% formic acid in water and 0.05% formic acid in acetonitrile). Compound purification was performed using an Agilent 1260 Infinity Preparative HPLC with a G1365D multiple wavelength detector set at λ= 210 nm and an Agilent Eclipse XDB-Phenyl column 21.2 x 100mm, 5 µm particle 12 size. High resolution mass spectrometry (HRMS) was performed on a Bruker Micro TOF mass spectrometer using (+)-ESI calibrated to sodium formate. Final compound purity of ≥ 95% was confirmed by LCMS analysis using both ELSD and UV (210 nm) detection.

Resin-bound Fmoc-L-Thr-OAllyl **3** (0.62 mmol/g) was prepared as previously described.[3]

General method for Peptide Coupling *(Compound **8** in Scheme S1 as example):* Fmoc protected resin **3** (100 mg, loading 0.62 mmol/g) was treated with a solution of 30% piperidine in DMF (2 mL) and shaken at rt for 15 min. The resin was drained and washed with DMF (× 3), MeOH (× 3) and DCM (× 3). The deprotection step was repeated. A solution of the corresponding amino acid (0.14 mmol, 2 eq.), DMF (0.29 mL), HCTU (0.5 M in DMF, 0.29 mL, 0.14 mmol, 2 eq.) and DIPEA (50 µL, 0.14 mmol, 4 eq.) was combined. After 1 min, the mixture was added to the resin. The resin was shaken for 30 min and then washed with DMF (× 3), MeOH (× 3) and DCM (× 3). The coupling step was repeated. An analytical sample was cleaved with TFA/$Et_3SiH/H_2O$ (95:1:4) followed by LCMS analysis to confirm coupling with corresponding amino acid.

The corresponding O-Allyl / N-Alloc protected heptapeptide DHP-resin **4** was washed with dry DCM (× 3). A solution of $Pd(PPh_3)_4$ (0.25 equiv) in dry DCM (2 mL) was added to the resin followed by phenylsilane (24 equiv). The resin was carefully shaken and the excess pressure was released several times until no increase in pressure was observed. The resin was then shaken at rt for 2 h, drained and washed with 0.5% DIPEA in DCM (× 5), 0.5% w/v sodium diethyl dithiocarbamate trihydrate in DMF (× 8), MeOH (× 3) and DCM (× 3) and dried under vacuum. An analytical sample was cleaved using TFA/$Et_3SiH/H_2O$ (95:1:4) followed by LCMS analysis to confirm complete *bis*-deprotection.

The O-Allyl / N-Alloc deprotected resin **5** was washed with DCM (× 3) and DMF (× 3) and then treated with a solution of DPPA (5.0 equiv) and DIPEA (10 equiv) in DMF (2 mL). The resin was shaken at room temperature overnight, drained and washed with DMF (× 3), MeOH (× 3) and DCM (× 3). An analytical sample was cleaved using TFA/$Et_3SiH/H_2O$ (95:1:4) followed by LCMS analysis to confirm complete cyclization. Resin **6** was treated with piperidine/DMF as described above to remove the P4 Fmoc group.

Fmoc SPPS was then continued to install the Fmoc-L-Thr(OtBu)-L-Dab dipeptide motif as described above using HCTU as coupling agent. Finally, the resin was *N*-acylated with a solution of octanoic acid (2 equiv), HCTU (2 equiv) and DIPEA (4 equiv) in DMF. The resin was shaken for 30 min and then washed with DMF (× 3), MeOH (× 3) and DCM (× 3). An analytical sample was cleaved with TFA/$Et_3SiH/H_2O$ (95:1:4) followed by LCMS analysis to confirm coupling.

The peptide was then cleaved from the resin by treatment with a solution of TFA/$Et_3SiH/H_2O$ (95:1:4) was added to the resin and the reaction vessel was shaken for 30 min at room temperature. The solvent was captured in a 20 mL vial, the resin was then washed with DCM (× 3), THF (× 3) and DCM (× 3) and all the fractions were combined to be evaporated under a stream of nitrogen. Crude peptide **8** was dissolved in 5 mL of acetonitrile/water (50/50, v/v) and freeze dried. Purification was performed using an Agilent 1260 Infinity Prep HPLC with an Agilent Eclipse XDB-Phenyl column (21.2 × 100 mm, 5 μm column, flow 20 mL/min), mobile phase A = 0.05% formic acid in water and B = 0.05% formic acid in acetonitrile, gradient 5 - 100% B over 20 min.

# Mass Spectrum Molecular Formula Report

**Analysis Info**

| | | | |
|---|---|---|---|
| Analysis Name | D:\Data\cooper\MCC_000748_002_RA4_01_9758.d | Acquisition Date | 9/14/2015 3:03:29 PM |
| Method | tune-wide_50ul_hystar_withcal_direct_medhighmass_2.m | Operator | a.piggott |
| Sample Name | MCC_000748_002 | Instrument / Ser# | micrOTOF 232 |
| Comment | Comments | | |

**Acquisition Parameter**

| | | | | | |
|---|---|---|---|---|---|
| Source Type | ESI | Ion Polarity | Positive | Set Nebulizer | 0.8 Bar |
| Focus | Not active | | | Set Dry Heater | 180 °C |
| Scan Begin | 100 m/z | Set Capillary | 4500 V | Set Dry Gas | 5.0 l/min |
| Scan End | 1500 m/z | Set End Plate Offset | -500 V | Set Divert Valve | Source |

**Generate Molecular Formula Parameter**

| | | | | | |
|---|---|---|---|---|---|
| Formula, min. | C51H90N14O12 | | | | |
| Formula, max. | C51H90N14O12 | | | | |
| Measured m/z | 545.343 | Tolerance | 10 ppm | Charge | 2 |
| Check Valence | no | Minimum | 0 | Maximum | 0 |
| Nirogen Rule | yes | Electron Configuration | even | | |
| Filter H/C Ratio | yes | Minimum | 0 | Maximum | 3 |
| Estimate Carbon | yes | | | | |

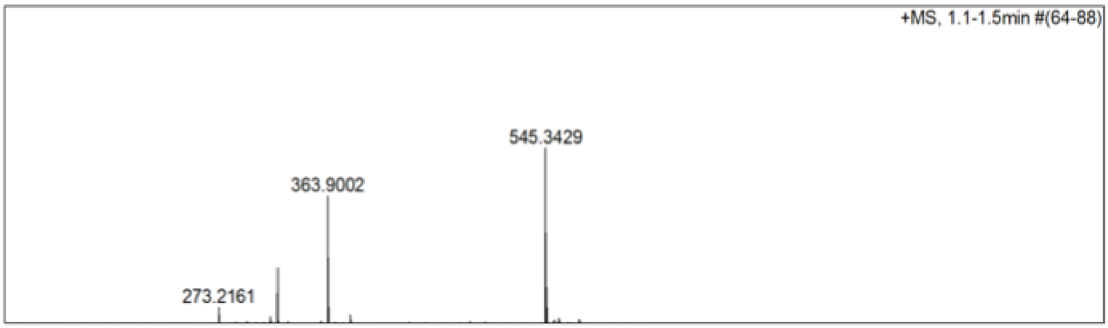


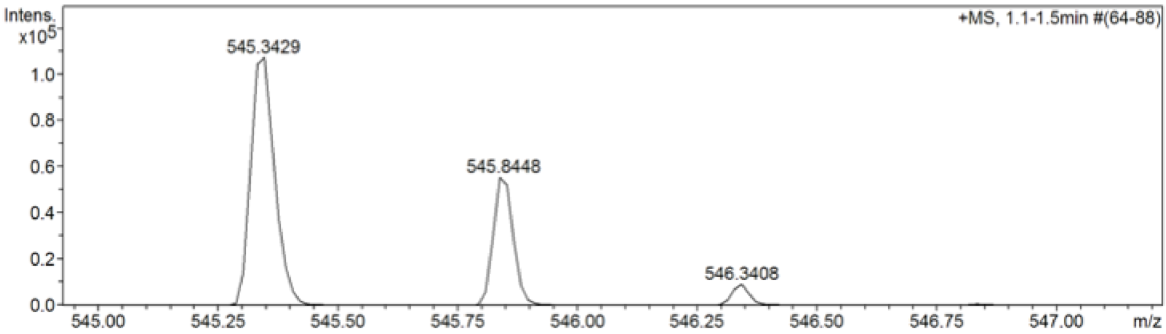


| Sum Formula | Sigma | m/z | Err [ppm] | Mean Err [ppm] | Err [mDa] | rdb | N Rule | e⁻ |
|---|---|---|---|---|---|---|---|---|
| C 51 H 90 N 14 O 12 | 0.076 | 545.3426 | -0.64 | 0.32 | -0.35 | 14.00 | ok | even |

**Figure S1:** HR-(+)-ESI-TOF-MS of the $[M+2H]^{2+}$ mass ion peak of compound **8**.

($C_{51}H_{90}N_{14}O_{12}$, calc $[M+2H]^{2+}$ = 545.3426)

## References:


1. Maatallah, M., M. Vading, M.H. Kabir, A. Bakhrouf, M. Kalin, P. Naucler, S. Brisse, and C.G. Giske, Klebsiella variicola is a frequent cause of bloodstream infection in the stockholm area, and associated with higher mortality compared to K. pneumoniae, *PLoS One*, **2014**, *9*, e113539. DOI 10.1371/journal.pone.0113539
2. Elliott, A.G., D. Ganesamoorthy, L. Coin, M.A. Cooper, and M.D. Cao, Complete Genome Sequence of Klebsiella quasipneumoniae subsp. similipneumoniae Strain ATCC 700603, *Genome Announc*, **2016**, *4*. DOI 10.1128/genomeA.00438-16
3. Gallardo-Godoy, A., C. Muldoon, B. Becker, A.G. Elliott, L.H. Lash, J.X. Huang, M.S. Butler, R. Pelingon, A.M. Kavanagh, S. Ramu, W. Phetsang, M.A.T. Blaskovich, and M.A. Cooper, Activity and Predicted Nephrotoxicity of Synthetic Antibiotics Based on Polymyxin B, *J Med Chem*, **2016**, *59*, 1068-1077. DOI 10.1021/acs.jmedchem.5b01593
